# The isometric horizontal push test correlates with jumping and sprinting performance among athletes and recreationally active controls

**DOI:** 10.5114/biolsport.2022.106158

**Published:** 2021-07-15

**Authors:** Antonio Dello Iacono, Carlos Holgado Lopez, Andisheh Bakhshi, Israel Halperin

**Affiliations:** 1 School of Health and Life Sciences, University of the West of Scotland, Glasgow, United Kingdom; 2 School of Computing, Engineering and Physical Science, University of the West of Scotland, United Kingdom; 3 School of Public Health, Sackler Faculty of Medicine, Tel Aviv University, Tel Aviv, Israel; 4 Sylvan Adams Sports Institute, Tel Aviv University, Tel Aviv, Israel

**Keywords:** Biomechanics, Football, Force, Musculoskeletal, Testing

## Abstract

The aim of this study was two-fold: (i) to examine the relationships between force outputs measured in the isometric horizontal push test (IHPT) and athletic performances; (ii) to compare IHPT outputs between football players and recreationally active controls. Thirty-two male subjects (football players, n = 16; university students, n = 16) completed the IHPT, countermovement jump (CMJ), standing long jump (SLJ), 5 m, 10 m and 20 m sprint tests, randomly across two testing sessions. Multivariate linear regression analysis was used to examine the relationships between IHPT outputs and athletic performances by accounting for the subjects’ athletic background. An independent sample t-test was used to compare the IHPT outputs between groups. Moderate to very strong linear relationships (r^2^ range: 0.16–0.56) were found between the IHPT and all athletic performances (all p < .026). Percent variance explained by the IHPT outputs after accounting for groups difference was 16%, 56%, 54%, 48% and 40% for CMJ height, SLJ distance, 5 m, 10 m and 20 m sprint performances, respectively. Compared to controls (6.18 ± 0.89 N/kg), football players (10.09 ± 1.57 N/kg) achieved greater IHPT force outputs (p < .001, Hedges’ *g* = 3.2, *large* ES). The IHPT is clearly correlated to horizontal and vertical athletic performances and can adequately distinguish between athletes and recreationally active controls based on their IHPT results. Future studies should examine the usefulness of the IHPT as a testing tool informing training prescription and performance monitoring practices.

## INTRODUCTION

Given the importance of force production in athletic activities, sport scientists and applied practitioners routinely assess force capabilities using a variety of physical tests [1, 2]. In particular, tests involving maximal voluntary isometric contractions [1, 3–5] are commonly implemented due to their high reliability [4, 6–9], ease to be administered [1, 4, 10], time efficiency [4] and minimal skill requirement [1, 4]. Two isometric tests frequently used in exercise and sport science settings are the isometric mid-thigh pull (IMTP) and the isometric squat (ISQT) tests [4–6, 9]. Both are reliable [4, 6–9, 11], are correlated with athletic performance indices, such as jump height (e.g., absolute and body mass relative gross peak force outputs across time intervals ranging from 50 ms to 300 ms) [11–14], sprint times over distances from 5 m to 20 m (e.g., absolute net peak force and peak rate of force development outputs as well as rate of force development variables expressed both as peak values and across time intervals ranging from 30 ms to 100 ms) [11–13, 15] and change of direction times (peak gross force and peak rate of force development outputs) [14, 15], and are able to distinguish between athletes of different competitive levels [11, 16–18].

Although the IMTP and ISQT tests are extensively studied and broadly implemented, both present two main limitations. First, both tests require subjects to assume an upright position, which closely resemble the body configurations of vertically-oriented tasks like jumping or weightlifting exercises, but of less relevance to tasks requiring forward orientation such as accelerations [18–22], sprints [20, 23] and horizontal jumps [20, 22]. Accordingly, when interpreting the relationships between force variables collected during IMPT and ISQT and athletic tasks performances, the specific body position and the reduced dynamic correspondence with horizontally-oriented tasks should be considered [24]. Indeed, numerous studies reported a large number of IMTP and ISQT outputs such as absolute, body mass relative or allometrically scaled gross peak forces, peak rate of force development and allometrically scaled rate of force development across time intervals ranging from 50 ms to 250 ms as strongly correlated with vertical jump height [25–27]. Likewise, strong correlations have been found between absolute gross and body mass relative gross peak force outputs in IMTP and ISQT tests and the one-repetition maximum loads in the squat [25, 28], power clean [25, 28], and deadlift exercises [29]. Conversely, absolute, body mass relative or allometrically scaled gross peak force outputs as well as rate of force development at 100 ms were only moderately correlated with short- and long-distance sprint times [11, 12, 15]. Second, the unique set up necessary to conduct these tests, requires a robust weightlifting cage securing the barbell as immobile as possible during their execution and a costly force plate, which are not accessible and affordable to many.

**FIG. 1:**
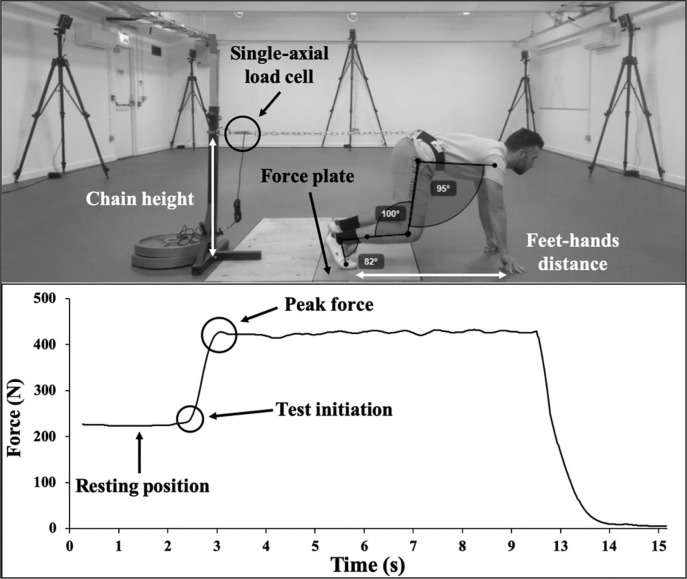
Isometric horizontal push test setup and associated force outputs. (Reprinted from Dello Iacono et al. The Isometric Horizontal Push Test: Test-Retest Reliability and Validation Study. Int J Sports Physiol Perform. 2019 Oct 11;1-4. doi:10.1123/ijspp.2019-0357.)

In view of the limitations of the IMTP and ISQT, Dello Iacono et al. [30] have recently developed a new isometric test – the Isometric Horizontal Push Test (IHPT) – that quantifies the horizontal component of the GRF produced during a maximal isometric effort in a crouched position such as that of a sprint start (Figure 1). The IHPT was validated against a force plate which is the gold standard method to assess isometric force. It was found reliable between days (intraclass correlation coefficient [ICC] = 0.99 and coefficient of variation [CV%] < 2.8%) and within a testing session (ICC ≥ 0.97 and CV% < 2%), and with a good degree of sensitivity (Smallest worthwhile change [SWC]: 29 N equal to 5.2%; Standard error of measurement [SEM]: 17 N [95% CI: 14, 20 N] equal to 3.1%) thus ensuring consistent and repeatable monitoring procedures of isometric force production. The IHPT can be easily administered using relatively cheap and portable equipment (strain gauge, metallic chain, weightlifting belt and carabineer hooks), it is time efficient and requires only a few trials to familiarize [30]. The setup position and the body orientation in the IHPT is partly similar to that an athlete assumes prior to performing horizontal jumps and short-sprint tasks, which presumes kinetic responses similar to these athletic tasks [18, 19, 31]. However, additional steps are currently required to establish the utility of the IHPT.

The primary aim of this study was to expand upon the work of Dello Iacono et al. [30] and examine the relationships between maximal isometric force outputs measured during the IHPT and vertically- and horizontally-oriented athletic tasks. A secondary aim was to determine whether differences in IHPT outputs exist between professional youth football players and recreationally active university students. We hypothesised that the IHPT outputs would strongly correlate with performances in athletic tasks as well as able to discriminate between athletes and non-athletic controls.

## MATERIALS AND METHODS

### Study design

A correlation study design was used to investigate the relationships between the IHPT outputs and athletic performances. One week before the experimental trials, subjects completed two familiarization sessions to become acquainted with the testing procedures. In particular, they were provided with instructions for the correct execution of all testing procedures, and completed two trials for each test following the same standardized warm-up implemented during the experimental sessions. Then, 48–72 hours after the familiarization sessions, subjects performed two testing sessions separated by a further 48 hours of recovery. On both occasions, subjects first completed a standardized warm-up followed by IHPT, countermovement jump (CMJ), standing long jump (SLJ) and 20 m linear sprint assessments, whose allocation across the two testing sessions (two tests per session) and order of execution within each session were randomly determined (www.random.org). Subjects were asked to refrain from completing strenuous physical activities and from consuming caffeine, alcohol, or any ergogenic substance two days prior and on the day of experimental sessions, respectively. Sessions were administered in the same facilities, at the same time of the day (3:00–6:00 PM), ambient temperature (22.1 ± 0.3°C) and relative humidity (61 ± 2%).

### Subjects

Sample size was calculated using *a priori* power analysis in the G*Power software (Heinrich-Heine-Universitat Dusseldorf, Germany). To this end, the summary results of a recent review article by Lum et al. [3] were used to compute the sample size calculation. Specifically, large correlations were reported between isometric force outputs expressed either as absolute values or relative to body mass and dynamic performances such as jump height, horizontal jump distance and sprint times. Accordingly, we used a correlation design with an α = 0.05, β = 0.2 and adequately powered (1 – β = 0.8), to detect strong linear relationships (r > 0.6) between the explanatory variable – IHPT output – and the outcome variables – athletic performances. This gave an estimated sample size of thirty subjects. Sixteen male university sport science students (21.3 ± 0.4 years; 76.1 ± 4.5 kg; 1.74 ± 0.11 m) and sixteen male professional youth football players (22.1 ± 0.8 years; 79.2 ± 7.2 kg; 1.78 ± 0.07), members of the U23 team of a Scottish Championship football club volunteered to participate to this study. University students were recreationally active, practiced concurrent outdoor (i.e., running and cycling) and gym-based (i.e., machine-based resistance training exercises) activities of moderate intensity at least two times a week (range: 2–3) for about 60 minutes each. At the time of study commencement, football players had just completed their preseason before the start of the 2019/20 competitive season and regularly trained between 5–7 times a week for about 90 minutes each session. All had at least six years (range: 6–8) of high-level football practice and three years (range: 3–5) of resistance training experience. Written informed consent was obtained after the subjects received an oral explanation of the purpose, benefits, and potential risks of the study. This study was in accordance with the Helsinki Declaration and approved by the Ethics Committee of the University in which the study was conducted.

### Procedures

#### Isometric Horizontal Push Test

Following a 10-min standardized warm-up including running drills and dynamic stretches, subjects completed three submaximal IHPT attempts equal to 60, 70 and 80% of their maximal effort. The IHPT assessments were conducted using the same setup (Figure 1) and procedures reported by Dello Iacono et al [30]. Heavy weight plates were laid on the base of the supporting stand, and nylon webbing straps were used to fix its upper end as to ensure that no movement occurred in the attached equipment during the test execution. Moreover, all subjects wore the same shoes across the testing days. Three maximal trials of 6 s were performed with 3 minutes of passive recovery between them. Strong verbal encouragement was provided by the same assessor during the trials [4]. The force outputs were collected by a portable strain gauge (Chronojump, Barcelona, Spain) sampling at 80 Hz. Data were then filtered through a 10 Hz Butterworth fourth order digital low pass filter as recommended by the manufacturer. The initiation of the push action was manually identified as the first time point corresponding to a force value 5 standard deviations (SD) greater than the mean value [4] recorded during the preparatory resting position lasting 2 s. The greatest force value at any point during the attempts was identified as the peak force. Peak force values were then normalized by body mass (N/kg) and used for data analysis.

#### Countermovement Jump

Vertical jump performance was assessed with a CMJ test. Starting position was stationary, erect, with knees fully extended and hands kept on the waist. Subjects squatted down to a self-selected height before beginning a forceful upward motion. Subjects were also instructed to avoid flexing hips, knees and ankles throughout the flight phase and at touchdown with the aim to limit any effect on jump height calculated according the flight time phase duration. Finally, they were instructed to jump as high as possible, and verbal encouragement was provided during the jumps. Subjects performed three attempts with passive recovery of 45 s between jumps, and the best result was recorded for data analysis. The jump height (cm) was measured with the Optojump apparatus (Optojump, Microgate, Bolzano, Italy).

#### Standing long jump test

Horizontal jump performance was assessed with a SLJ test [32]. Subjects stood behind a take-off line marked on the ground, with feet slightly apart. Then, they pushed off the ground vigorously and jumped forward as far as possible. A two-foot take-off and landing were used with swinging of the arms. SLJ performance was measured with a standard measuring tape as the jump distance (cm) from the take-off line to the nearest point where the back of the heel landed. Three attempts were performed with passive recovery of 60 s between jumps, and the best result recorded for data analysis.

#### Sprint test

Sprint performance was evaluated with a 20 m all-out run [31]. Subjects were asked to assume a three-point start position, with the plant hand placed 0.3 m before the starting line. During the attempts, strong verbal encouragement was provided. Sprint times was recorded using timing gates (Witty system, Microgate, Bolzano, Italy) placed at start line and on the 5 m, 10 m and 20 m lines, approximately 0.5 m above the ground. The test was performed three times, separated by 2 minutes of passive recovery. The best performances over 5 m, 10 m, 20 m across the three trials were recorded and used for analysis.

### Statistical Analyses

The intra-day reliability of the IHPT and dynamic performances were examined by calculating the CV% [33] and the Intra-class Correlation Coefficient (ICC_3,1_). A CV < 10% was considered a cut-off value for good reliability [34]. ICC values were interpreted as unacceptable < 0.5, 0.6 > poor ≥ 0.5, 0.7 > questionable ≥ 0.6, 0.8 > acceptable ≥ 0.7, 0.9 > good ≥ 0.8 and excellent ≥ 0.9 [35]. The assumptions for applying multivariate linear regression modeling method were tested. Absolute skewness and kurtosis values smaller than 2 also served as indications of normality and lack of obvious outliers. Assumption of homoscedasticity was confirmed by visually inspecting the scatterplot of fitted values and residuals of the fitting model. Multivariate regression modelling was applied to estimate changes in outcome variables – athletic performances – as a factor of the continuous covariate IHPT and the categorical covariate group as follow:

*y*_i_ = *β*_0_ + *β*_1_*IHPT* + *β*_2_*group* + *ε*_i_

Where *ε*_i_ denotes changes in the outcome variable; β_0_ is the coefficient of the intercept when *IHPT* = 0, which was not meaningfully interpreted and only included for improvement of the model fit; β_1_ is the coefficient of the covariate *IHPT* when > 0; β_2_ is the coefficient of the covariate *group*; *ε*_i_ is the error that represents the deviation of the data points from the regression line. The covariate *group* was treated as a binary variable with two categories, athletes and controls, and the category controls was considered as reference in the regression model. This means that the interpretations of the estimate parameters of the category athletes were made with respect to the category controls.

To examine the mutual relationships between the outcome variables – athletic performances – and the explanatory variable *IHPT* when the covariate *group* is held constant, we calculated the coefficient of partial determination (partial r^2^). Qualitative interpretation of partial r^2^ outcomes was reported according to Hokpins [36], with values between 0–0.01, 0.01–0.09, 0.09–0.25, 0.25–0.49, 0.49–0.81, 0.81–1 and equal to 1 (All intervals are of the form r^2^_low_ ≤ r^2^ < r^2^_high_) for trivial, small, moderate, strong, very strong, nearly perfect and perfect relationships, respectively. Differences between groups in IHPT outputs were analysed using an independent-sample t-test. Significance was at p < 0.05. 95% CI and Hedges’ *g* effect size (ES) [37] are reported alongside the p values. Analyses were performed in Jamovi statistics software (Version 1.2.27.0).

## RESULTS

**FIG. 2:**
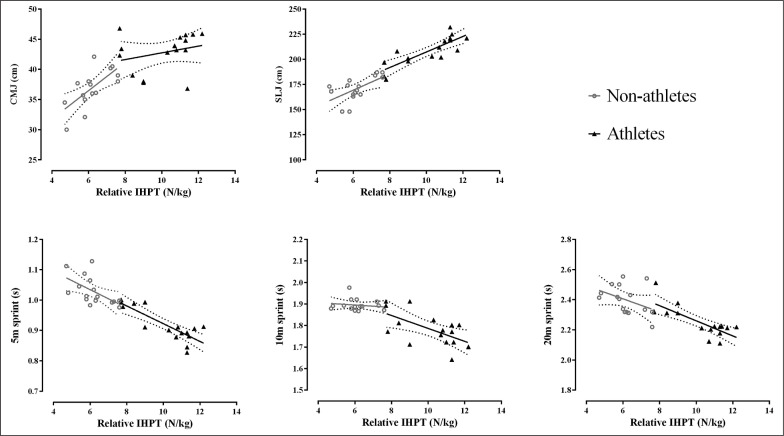
Illustrates the linear relationships between the IHPT outputs and the dynamic performances. Individual data points are presented as well as linear regression lines with 95% CI for both groups. IHPT = isometric horizontal push test; CMJ = countermovement jump; SLJ = standing long jump.

Descriptive statistics for all continuous variables of both groups are presented as mean ± SD and 95% confidence interval (95% CI) in Table 1. The CV% and ICC scores of the intra-day IHPT, CMJ, SLJ, 5 m, 10 m and 20 m scores were 3.2% (95% CI: 2.8, 3.6) and 0.89 (95% CI: 0.85, 0.93), 4.5% (95% CI: 4, 5) and 0.77 (95% CI: 0.74, 0.80), 3.9% (95% CI: 3.4, 4.3) and 0.81 (95% CI: 0.76, 0.86), 1.2% (95% CI: 1, 1.4) and 0.91 (95% CI: 0.83, 0.98), 2.3% (95% CI: 1.9, 2.7) and 0.88 (95% CI: 0.81, 0.95) 2.4% (95% CI: 1.9, 2.9) and 0.76 (95% CI: 0.72, 0.80), demonstrating good absolute and acceptable to excellent relative reliability, respectively. Results indicated moderate to very strong linear relationships between the IHPT outputs and all dynamic performances (all p < .026) (Table 2). Plots of the linear regression analyses between the IHPT outputs and the dynamic performances can be observed in Figure 2. The percent variance explained by IHPT outputs after accounting for groups’ difference was 16% in CMJ, 56% in SLJ, and 54%, 48% and 40% in 5 m, 10 m and 20 m sprint performances, respectively. Significant differences between groups were identified for IHTP, with football players (10.09 ± 1.57 N/kg) achieving greater (95% CI: 3, 4.84; *t* = 8.72; p < .001; Hedges’ *g* = 3.2, *large* ES) force outputs than the controls (6.18 ± 0.89 N/kg).

**TABLE 1:** Descriptive statistics (mean ± SD and 95% CI) of isometric force and dynamic performances variables of both groups.

Test	Athletes	Controls
IHPT (N/kg)	10.09 ± 1.57 (9.32, 10.90)	6.18 ± 0.89 (5.7, 6.66)
CMJ (cm)	42.8 ± 3.2 (40.6, 44.3)	36.9 ± 3.1 (35.4, 38.4)
SLJ (cm)	208.1 ± 14.6 (201.0, 215.0)	170.8 ± 11.8 (164.5, 177.1)
5m sprint (s)	0.919 ± 0.055 (0.890, 0.948)	1.029 ± 0.046 (1.004, 1.054)
10m sprint (s)	1.784 ± 0.077 (1.742, 1.824)	1.894 ± 0.028 (1.879, 1.908)
20m sprint (s)	2.255 ± 0.100 (2.201, 2.308)	2.398 ± 0.095 (2.347, 2.448)

IHPT: isometric horizontal push test; CMJ: countermovement jump: SLJ: standing long jump; CI: confidence intervals; N: Newton

**TABLE 2:** Multivariate regression modelling outputs between IHPT scores (N/kg) and athletic background and dynamic performances.

Performance	Parameter estimate (95% CI)	SE	p value	partial r^2^
CMJ (cm)	0.99 (0.13, 1.85)	0.42	.026	.16
**A**–**C**	2.01 (-1.98, 5.99)	1.95	.310	
SLJ (cm)	7.81 (5.18, 10.44)	1.29	< .001	.56
**A**–**C**	6.69 (-5.48, 18.86)	5.95	.270	
5m sprint (s)	-0.029 (-0.039, -0.019)	0.005	< .001	.54
**A**–**C**	-0.005 (-0.053, 0.042)	0.023	.813	
10m sprint (s)	-0.024 (-0.038, -0.009)	0.007	.002	.48
**A**–**C**	-0.016 (-0.051, 0.083)	0.033	.629	
20m sprint (s)	-0.048 (-0.071, 0.025)	0.011	< .001	.40
**A**–**C**	-0.047 (-0.152, 0.058)	0.051	.368	

IHPT: isometric horizontal push test; N: Newton; kg: kilograms; CI: interval of confidence; SE: standard error; A: athletes; C: control; CMJ: countermovement jump: SLJ: standing long jump; cm: centimetre. Note: positive A–C mean difference scores in CMJ and SLJ, and negative A–C mean difference scores in all sprint tests mean better performances in the A group compared to the C group.

## DISCUSSION

The aim of this study was two-fold. The primary was to examine the relationships between isometric force outputs collected during the IHPT and athletic performances measured with common field tests. A secondary aim was to determine whether differences in IHPT outputs exist between professional youth football players and recreationally active university students. Moderate to very strong linear relationships (r^2^ range: 0.16–0.56) were found between the IHPT and all athletic performances (all p < .026). Also, compared to controls (6.18 ± 0.89 N/kg), football players (10.09 ± 1.57 N/kg) achieved greater IHPT force outputs (p < .001, Hedges’ *g* = 3.2, *large* ES).

Moderate to very strong linear relationships were detected between the IHPT outputs and both jump and sprint performances confirming the association between maximal isometric force outputs and athletic performances. By comparing the regression model fit coefficients between the IHPT outputs and the athletic performances two interesting outcomes emerged. First, IHPT outputs explain variations in both SLJ and sprint performances (partial r^2^ range: 0.40–0.56) to a greater extent and with less margin of error than the CMJ (partial r^2^ = 0.16) (Table 2). This suggests the IHPT as better suited to estimate performance of tasks in which the body is horizontally propelled and require horizontal GRF. This finding is not surprising and can be explained by the principle of dynamic correspondence [24]. In particular, while the CMJ is performed along the vertical axis and relies primarily on vertical GRF, both the SLJ and sprints are horizontal in nature, with greater antero-posterior GRF demands. In this context, Kugler and Janshen [38] have reported that the body kinematics during a specific task is highly correlated (*r* = 0.93) to the vector of the GRF relative to the body, and more importantly the dynamic correspondence is the key determinant for the ability to express force along a specific direction. Therefore, the biomechanical similarity between the IHPT and both SLJ and sprints can assist explaining the stronger relationships with these tasks than the CMJ.

Another interesting finding concerns the pattern of variance explained by IHPT outputs for changes in sprint performances, which decreased as the sprint distance increased (partial r^2^ equal to 0.54, 0.48 and 0.4 for 5 m, 10 m and 20 m, respectively). This finding can be explained by the greater biomechanical similarities between IHPT and the initial acceleration phase (i.e., 5 m) of the sprint. During this phase, the body leans forward in a crouched position with an overall geometric configuration and specific joints’ configurations more similar to those assumed during the IHPT execution. Conversely, such dynamic correspondence is lost during the late stages of the sprint (i.e., 10 m and 20 m) as the body progressively moves into an upright position [31, 39]. These changes of body configuration couple with characteristic kinetic patterns. Specifically, a concurrent and progressive shift of the resultant GRF vector from a horizontal into a more vertical direction occurs as the sprint distance increases. Computational and observational studies have confirmed this assumption by examining the changes in the ratio of forces (i.e. horizontal component of the GRF vector expressed as a percentage of the total GRF vector magnitude) across consecutive contact phases during sprint trials of same distances as those investigated in this study [18, 39–41]. Moreover, it is plausible that the discrepancy across the relationships is associated with the time available to develop force during sprint tasks of different distances. In fact, foot contact time during the acceleration phase of sprinting is about 300 ms and progressively decreases to 90–100 ms at top speed [42]. The length of time for force production during the acceleration phase is sufficient to achieve high absolute levels of force, potentially similar to the peak force outputs exploited during the IHPT (Figure 1). In contrast, it is unlikely that maximal horizontal force outputs can be achieved in shorter intervals of time as running speed increases [43]. The findings of this study confirm the meaningful relationships between isometric horizontal force production and horizontally oriented athletic performances.

The results of this study revealed between-group differences in IHPT outputs as football players achieved greater scores than the controls (10.09 ± 1.57 vs 6.18 ± 0.89 N/kg, *large* ES). This finding aligns to what is generally reported in the sport literature whereby maximal isometric force levels can distinguish athletes from recreationally active populations [44]. Different force outputs between groups may be largely explained by the exposure to high-intensity practice including accelerations, decelerations, sprints and changes of direction that football players and not controls routinely perform during training and competition. In fact, these locomotive demands represent part of the conditioning stimuli underpinning physical development and performance maintenance [45], and the likely discriminant factor contributing to the superior force levels of football players than controls observed in this study. We note that the between-group differences in force levels should be interpreted further by considering the relationships between IHPT outputs and all athletic performances after accounting for the background of the subjects of this study. While IHPT outputs can be used to estimate athletic performances likewise across athletes and controls, we observed a consistent pattern, from which superior athletic performances are expected for each unit increase in IHPT among athletes compared to controls (Table 2 and Figure 2). Consistent with the contemporary literature [2, 3, 11–16, 25–29, 46, 47], athletes who perform better in dynamic tasks are also reported to produce higher isometric force levels. This is a finding of practical value as it provides indication that horizontal force capabilities measured through the IHPT represent a relevant physical determinant for participation in sport at elite level.

This study has a few limitations worthy of discussion. Firstly, the results can only be generalized to male athletes with a team sport background. Future research would benefit from testing other populations like females and athletes competing in individual sport disciplines. Second, although a priori power analysis was conducted to determine the necessary sample size, only sixteen U23 professional football players participated to this study which narrows what can be concluded with regard to younger and less trained or professional adult high-level football players. Third, this study adopted a correlation design aimed at investigating the relationships between the IHPT force outputs and dynamic performances. Future longitudinal interventions are then warranted to determine if increases in IHPT force production translate to improvements in performance in athletic tasks. Finally, we used only peak force outputs as primary outcome without evaluating specific force-time variables which may could be used to investigate the sensitivity of this test as a monitoring tool.

## CONCLUSIONS

This study identified that isometric peak force measured during the IHPT is related to measures of dynamic performance in both professional youth football players and recreationally active university students. Moreover, football players produced greater IHPT outputs compared to recreationally active controls. Sport scientists and practitioners should consider implementing the IHPT to measure maximal horizontal force production capabilities in a simple and time efficient manner. For example, the IHPT could be used to measure isometric force capabilities alongside the assessment of dynamic performances or when the latter are not feasible or impractical. Moreover, the ease to administer and immediate interpretation of the IHPT results, make it a suitable approach also to monitor large groups of athletes.
